# Association between systemic immune-inflammation index and sleep disorder among US adults: A cross-sectional study based on NHANES 2005 to 2020

**DOI:** 10.1097/MD.0000000000045186

**Published:** 2025-10-24

**Authors:** Yang Yang, Defeng Chen, Xuhui Dong, Bei Li, Mengxin He, Jiabao Li, Yaokai Xu, Yueyu Liang

**Affiliations:** a Department of Gastrointestinal, Hernia and Enterofistula Surgery, The People’s Hospital of Guangxi Zhuang Autonomous Region, Nanning, Guangxi, China; b School of Nursing, Youjiang Medical University for Nationalities, Baise, Guangxi, China; c School of Nursing, Guangxi University of Chinese Medicine, Nanning, Guangxi, China.

**Keywords:** cross-sectional study, NHANES sleep disorder, restricted cubic spline, Systemic Immune-Inflammation Index

## Abstract

The relationship between the Systemic Immune-Inflammation Index (SII) and sleep disorders remains poorly characterized. This cross-sectional study investigated this association utilizing data from the National Health and Nutrition Examination Survey spanning 2005 to 2020. A total of 42,150 participants were included, of whom 25.34% reported sleep disorders. The association between SII and sleep disorders was evaluated using multivariable logistic regression models and restricted cubic spline analysis. In unadjusted models, a significant positive association was observed; compared with the lowest SII quartile (Q1), the highest quartile (Q4) had 20.32% higher odds of sleep disorders (OR = 1.2032; 95% CI: 1.1309–1.2801; *P* < .0001). After adjustment for age, sex, and race, the association persisted (OR = 1.1118, 95% CI: 1.0425–1.1858, *P* = .0013). In more fully adjusted models (including body mass index, smoking, education, poverty-income ratio, diabetes, cardiovascular disease, and hypertension), the linear association was not statistically significant. Restricted cubic spline analyses showed a U-shaped association. Subgroup analyses indicated statistical effect modification by sex and by hypertension status. Overall, higher SII was associated with increased odds of reporting sleep disorders in partially adjusted models; however, in the fully adjusted models the association was not statistically significant (*P* > .05), suggesting that it may largely reflect shared correlates rather than an independent effect. Future longitudinal studies are warranted to clarify temporal relationships and to assess the incremental clinical utility of SII in sleep health.

## 1. Introduction

Sleep disorders are defined as a group of symptoms that manifest when individuals are unable to obtain sufficient and quality sleep due to various factors, thereby affecting their physical health, mental state, and social functioning.^[1]^ The advent of modernity and the attendant changes in lifestyle have resulted in a steady rise in the prevalence of sleep disorders on an annual basis, thus giving rise to a major global public health concern.^[2]^ According to reports from the World Health Organization, approximately one-third of the global population is affected by various forms of sleep problems.^[3]^ A meta-analysis demonstrated an association between sleep disorders and elevated levels of systemic inflammation.^[4]^ In the study by Güneş and colleagues, the Systemic Immune-Inflammation Index (SII) was higher in patients with polysomnography-confirmed obstructive sleep apnea (OSA), with the degree of increase aligning with the severity of the disorder.^[5]^ The repercussions of sleep disorders on individuals’ daily lives and work efficiency are well-documented, and they may also lead to a range of health issues, including cardiovascular disease (CVD), diabetes mellitus (DM), depression, and anxiety disorders.^[6]^ Moreover, confirming specific sleep disorders typically requires specialized assessments such as questionnaire-based evaluations and polysomnography,^[7]^ which limits population-level evaluation of sleep health. Our study examines the association between sleep disorders and the SII, not solely sleep-disordered breathing. Self-reported sleep disorders have been widely used in National Health and Nutrition Examination Survey (NHANES) and in epidemiologic and outcomes research, offering good comparability and reproducibility.^[8,9]^ Evidence indicates that self-reported sleep disorders are associated with an increased risk of all-cause mortality,^[10]^ therefore, self-assessment of sleep status has important value for public health management. However, because the analysis used self-reported measures of sleep disorders and did not conduct a pooled analysis across different disorder categories, this is a limitation of the present study.

The SII is an important indicator for assessing the body’s immune and inflammatory status, primarily reflecting systemic inflammation through parameters such as white blood cell count, neutrophil count, and platelet count.^[11]^ Recent studies have shown that SII levels are closely related to the occurrence and progression of various diseases, including CVD, DM, and sleep disorders.^[12,13]^ In addition, as people gradually discover the potential connection between SII and sleep disorders.^[14]^ the relationship between SII and sleep disorders deserves widespread societal attention.^[15]^

A high SII typically indicates the presence of chronic low-grade inflammation, and this persistent immune activation may affect the brain’s sleep regulation mechanisms through various pathways.^[16]^ For example, inflammatory mediators such as cytokines can directly act on the brain’s sleep centers, disrupting the normal sleep–wake cycle.^[17]^ Moreover, chronic inflammation may lead to excessive activation of the sympathetic nervous system, further exacerbating feelings of anxiety and stress, thereby impairing sleep quality.^[18]^ Accordingly, inflammation and sleep disorders likely have a biologically plausible bidirectional relationship. On this basis, our study aims to more precisely characterize the association and potential interactions between SII and sleep disorders.^[19]^ Considering the availability and coherence of the data over time, the present study defined sleep disorders using SLQ050 (“Ever told doctor had trouble sleeping?”) thereby indexing subjective sleep complaints with broader coverage. To the best of the author’s knowledge, no studies have utilized samples from the period 2005 to 2020 to investigate the relationship between SII and sleep disorders to the best of the author’s knowledge. This cross-sectional study was conducted using data from the NHANES from 2005 to 2020. The NHANES data were approved by the National Center for Health Statistics Research Ethics Review Board, and all participants provided informed consent.^[20]^

## 2. Materials and methods

### 2.1. Study population and date

**Figure 1.**
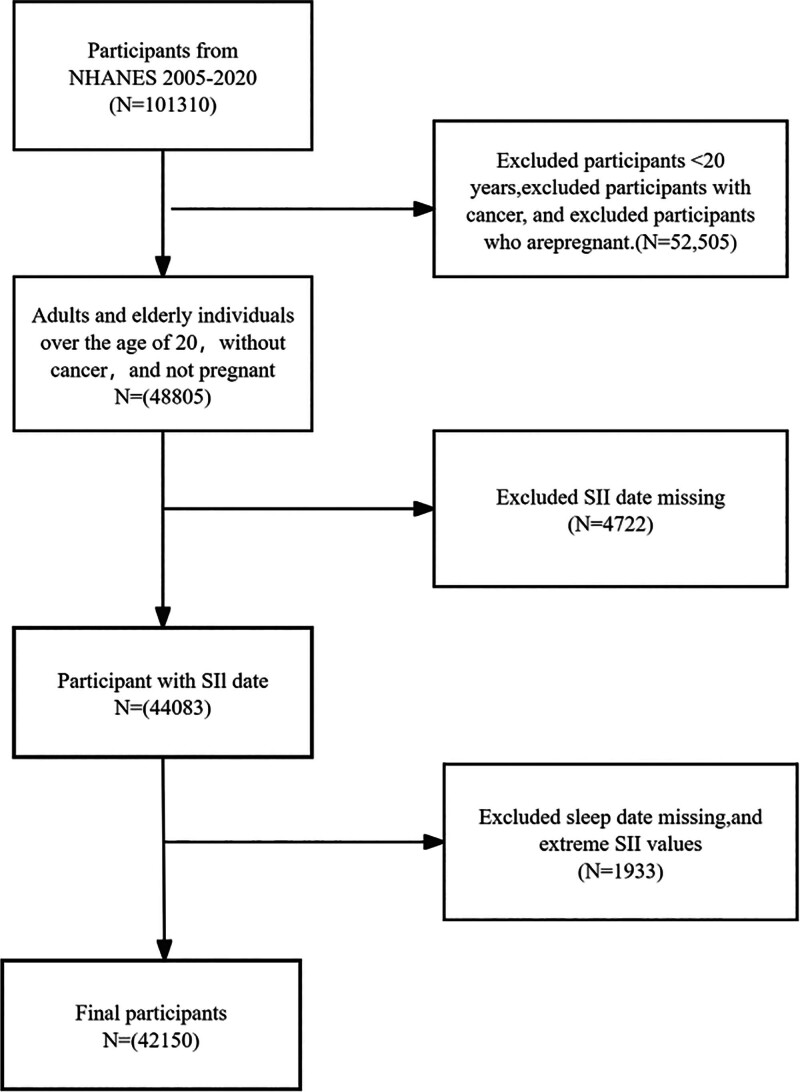
Flowchart of participants selection. NHANES = National Health and Nutrition Examination Survey, SII = Systemic Immune-Inflammation Index.

The NHANES is sponsored by the Centers for Disease Control and Prevention and is a population-based cross-sectional study designed to assess the health and nutritional status of both adults and children in the United States. It is conducted through the National Center for Health Statistics of the Centers for Disease Control and Prevention. In this study, data from 8 2-year cycles of NHANES (2005–2020) were combined, resulting in a total of 101,310 eligible participants and the survey was a cross-sectional study of U.S. residents aged 20 years or older. According to the objectives of this study, the data were screened based on the following exclusion criteria: participants under 20 years of age; participants diagnosed with cancer; pregnant participants; participants with missing SII values; participants lacking sleep-related data; participants exhibiting extreme SII values. After manual data screening, 42,150 participants were ultimately selected for analysis. The process of sample selection is illustrated in Figure 1.

### 2.2. Definition of a variable

#### 2.2.1. SII

Lymphocyte, neutrophil, and platelet counts were obtained by performing a complete blood count on blood specimens with a Beckman Coulter automated blood analyzer in an Mobile Examination Center and were expressed as ×10^3^ cells/µL. According to previous studies,^[14]^ SII was defined as follows: SII = platelet count × neutrophil count/lymphocyte count.

### 2.3. Sleep disorder

In response to the question {Have you/Has SP} ever told a doctor or other health professional that {you have/s/he has} trouble sleeping? Sleep disorder was self-reported.^[2]^

### 2.4. Covariables

Covariates included age, race, gender, smoking status, educational attainment, poverty-income ratio (PIR), educational level, CVD, and hypertension (HTN). Given that body mass index (BMI) and DM can, from both physiological and epidemiological perspectives, simultaneously influence the occurrence of sleep disorders and levels of the SII,^[21,22]^ we treated BMI and DM as confounders in our analyses.

### 2.5. Statistical methods

DecisionLinnc.1.0. (Hangzhou, China) November 2023. DecisionLinnc is a platform that integrates multiple programming language environments and enables data processing, data analysis, and machine learning through a visual interface.^[23]^ Group comparisons between participants with and without sleep disorders were conducted using the χ² test and the *t* test. Using a logistic regression model to test the linear relationship between SII and sleep disorders. The crude model 1 was adjusted for no covariates. Model 2 was a minimally-adjusted model that included age, gender, and race. Model 3 adjusted for the PIR, education level, BMI, smoking history, DM statuses, CVD statuses, and HTN statuses. Subgroup analysis was employed to investigate the relationship between the SII and sleep disorders among American adults of different ages, genders, PIR, educational levels, BMI, smoking statuses, DM statuses, CVD statuses, and HTN statuses. Using restricted cubic spline (RCS) to explore the nonlinear relationship between SII and sleep disorders.

## 3. Results

### 3.1. Characteristics of the participants

In total, 42,150 participants were enrolled in this study. The overall mean age of the surveyed population is 48.3667 ± 17.1809 years. Table 1 presents the characteristics of the participants. We categorized the participants (n = 42,150) into 2 groups: the “sleep disorder” group and the “no sleep disorder” group. The sample distribution is approximately balanced between females (51.24%) and males (48.76%). Among participants without sleep disorders, the mean SII was 473.0576 ± 210.786; among those who self-reported a sleep disorder, the mean SII was 490.3238 ± 220.9254. Racial composition is varied, with 36.87% non-Hispanic White, 23.64% non-Hispanic Black, 15.35% Mexican American, and 10.35% other Hispanic, with 13.79% categorized as other race. In terms of education, 23.29% of participants had less than a high school education, 23.26% had a high school education, and 53.45% had education above high school level. Smoking status shows that 57.52% of the individuals have never smoked, 22.38% are former smokers, and 20.10% are current smokers. CVD prevalence is noted at 7.51%, and DM is present in 15.23% of the population. HTN is reported in 34.32% of individuals. Furthermore, in total, 10,680 participants self-reported a sleep disorder (Table 1).

**Table 1:** Basic participant characteristics based on the Systemic Inflammation Index of American adults.

Variable names	Level	Overall	No sleep disorder	Sleep disorder	*P*
n		42150	31470 (74.66)	10680 (25.34)	
Age (yr)		48.3667 ± 17.1809	47.1705 ± 17.4049	51.8915 ± 15.9919	<.0001
SII		477.4325 ± 213.5301	473.0576 ± 210.786	490.3238 ± 220.9254	<.0001
Poverty-income ratio		2.4547 ± 1.5733	2.4632 ± 1.5659	2.4298 ± 1.5948	.0582
BMI		29.425 ± 7.1145	28.8745 ± 6.7177	31.0474 ± 7.9541	<.0001
Gender (%)	Female	21599 (51.24)	15395 (48.92)	6204 (58.09)	<.0001
	Male	20551 (48.76)	16075 (51.08)	4476 (41.91)	
Race (%)	Mexican American	6468 (15.35)	5322 (16.91)	1146 (10.73)	<.0001
	Other Hispanic	4364 (10.35)	3307 (10.51)	1057 (9.90)	
	Non-Hispanic White	15541 (36.87)	10729 (34.09)	4812 (45.06)	
	Non-Hispanic Black	9964 (23.64)	7456 (23.69)	2508 (23.48)	
	Other race	5813 (13.79)	4656 (14.80)	1157 (10.83)	
Education (%)	Less than high school	9816 (23.29)	7568 (24.05)	2248 (21.05)	<.0001
	High school	9805 (23.26)	7265 (23.09)	2540 (23.78)	
	Above high school	22529 (53.45)	16637 (52.87)	5892 (55.17)	
Smoking status (%)	Never	24245 (57.52)	19028 (60.46)	5217 (48.85)	<.0001
	Former	9434 (22.38)	6580 (20.91)	2854 (26.72)	
	Current	8471 (20.10)	5862 (18.63)	2609 (24.43)	
Cardiovascular disease (%)	No	38984 (92.49)	29643 (94.19)	9341 (87.46)	<.0001
	Yes	3166 (7.51)	1827 (5.81)	1339 (12.54)	
Diabetes (%)	No	35731 (84.77)	27415 (87.11)	8316 (77.87)	<.0001
	Yes	6419 (15.23)	4055 (12.89)	2364 (22.13)	
Hypertension (%)	No	27683 (65.68)	22319 (70.92)	5364 (50.22)	<.0001
	Yes	14467 (34.32)	9151 (29.08)	5316 (49.78)	

The results table presents the mean ± standard deviation for each continuous variable, along with frequencies and percentages for categorical variables. The *P*-values were derived from comparisons of 2 group means (*t* test) or comparisons of means across 3 or more groups (analysis of variance, ANOVA).

Association between SII and sleep disorder.

SII = Systemic Immune-Inflammation Index.

### 3.2. Association between SII and sleep disorder

**Figure 2.**
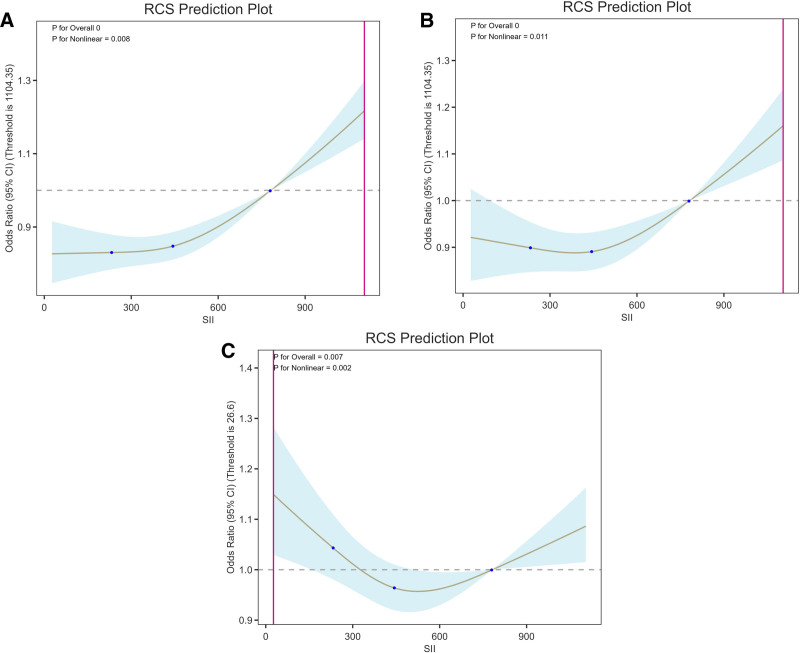
Restricted cubic spline transformation of the nonlinear relationship between sleep disorders and the Systemic Inflammation Index. Model A: no adjustment for covariates. Model B: adjusted for age, gender, and race. Model C: adjusted for age, gender, race, education level, PIR, BMI, smoking, diabetes, coronary heart disease, and hypertension. BMI = body mass index, PIR = poverty-income ratio.

To address negligible effect values, SII was divided by 100 to rescale the effect estimates. Table 2 shows the relationship between SII and sleep disorders. The results of the multivariate regression analysis between SII/100 and sleep disorders are shown in Table 2. In the unadjusted model (Model 1), SII/100 was positively associated with sleep disorders (OR = 1.0381; 95% CI, 1.0276–1.0487; *P* < .0001). In the model adjusted for age, sex, and race (Model 2), the association remained significant but was attenuated (OR = 1.0235; 95% CI, 1.0127–1.0343; *P* < .0001). In the fully adjusted model (Model 3), the association between SII/100 and sleep disorders was no longer significant (OR = 0.9970; 95% CI, 0.9861–1.0080; *P* = .5928), indicating that after comprehensive adjustment for covariates, the effect of SII as a continuous variable on sleep disorders is not significant. The nonlinear relationship between SII and sleep-related disorders was explored using RCS. The RCS plot (Fig. 2) illustrates the nonlinear relationship between the SII and sleep-related disorder. The results of the RCS analysis are shown in Figure 2. For the SII indicator, the analysis revealed a U-shaped nonlinear relationship with the risk of developing sleep disorders (nonlinear *P* < .01), with a significantly increased risk observed at both higher and lower levels of SII (Fig. 2C).

**Table 2:** The relationship between the Systemic Inflammation Index and sleep disorders.

	Crude model (Model 1)	Model 2	Model 3
	OR (95% CI) *P*-value	OR (95% CI) *P*-value	OR (95% CI) *P*-value
SII/100	1.0381 (1.0276.1.0487) *P* < .0001	1.0235 (1.0127–1.0343) *P* < .0001	0.9970 (0.9861–1.0080) *P* = .5928
SII quartiles			
Q1	1	1	1
Q2	1.0458 (0.9819.1.1138) *P* = .1636	1.0309 (0.9665–1.0997) *P* = .3553	0.9859 (0.9228–1.0534) *P* = .6751
Q3	1.0884 (1.0223.1.1588) *P* = .0081	1.0376 (0.9726–1.1069) *P* = .2633	0.9500 (0.8889–1.0153) *P* = .1307
Q4	1.2032 (1.1309.1.2801) *P* < .0001	1.1118 (1.0425–1.1858) *P* = .0013	0.9557 (0.8941–1.0215) *P* = .1822

Model 1: no adjustment for covariates. Model 2: adjusted for age, gender, and race. Model 3: adjusted for age, gender, race, education level, PIR, BMI, smoking, diabetes, coronary heart disease, and hypertension.

BMI = body mass index, PIR = poverty-income ratio.

### 3.3. Subgroup analyses

In this analysis, we assessed the stability of the association between SII and sleep-disorder status and explored potential heterogeneity across population subgroups; the results are shown in Table 3. In stratified analyses by gender and HTN status, the *P*-values for interaction were .0236 and .0099, indicating that the association between SII and sleep-disorder status differed across these subgroups. By contrast, the interaction terms for age, race, and educational level were all > 0.05, providing little evidence of heterogeneity by these characteristics. Overall, the association between SII and sleep-disorder status appeared generally consistent across groups, with possible subgroup differences by gender and HTN status (Table 3).

**Table 3:** The subgroup analysis of the relationship between SII/100 and sleep disorders.

Subgroup	SII/100 OR [95% CI]	*P* for interaction
Gender		.0236
Female	1.02 (1.01–1.03)	
Male	1.05 (1.03–1.06)	
Race		.1806
Mexican American	1.03 (0.99–1.06)	
Other Hispanic	1 (0.96–1.03)	
Non-Hispanic White	1.04 (1.02–1.05)	
Non-Hispanic Black	1.03 (1–1.05)	
Other race	1.05 (1.02–1.08)	
Education		.3504
Less than high school	1.03 (1–1.05)	
High school	1.05 (1.03–1.07)	
Above high school	1.04 (1.02–1.05)	
PIR		.4088
<1.3	1.03 (1.01–1.05)	
1.3–3.5	1.04 (1.02–1.05)	
>3.5	1.05 (1.03–1.07)	
BMI		.6508
18.5 ≤ BMI < 24	1.03 (1.01–1.06)	
<18.5	1.07 (0.99–1.17)	
≥24	1.03 (1.02–1.05)	
HTN		.0099
No	1.05 (1.03–1.06)	
Yes	1.02 (1–1.03)	
DM		.8767
No	1.04 (1.02–1.05)	
Yes	1.03 (1.01–1.06)	

BMI = body mass index, HTN = hypertension, PIR = poverty-income ratio.

## 4. Discussion

In the baseline characteristics analysis, participants with sleep disorders were notably older and included a higher proportion of women, consistent with prior epidemiologic patterns of higher prevalence among females.^[24]^ Potential mechanisms may involve sex hormone levels, pregnancy history, age-related metabolic changes, and lifestyle factors. In addition, non-Hispanic White individuals accounted for a relatively higher proportion among those with sleep disorders, suggesting that racial/ethnic background may influence the likelihood of sleep-disorder status, potentially reflecting differences in dietary habits and metabolic profiles. HTN, DM, and coronary heart disease were also more concentrated in the sleep-disorder group. As these conditions are commonly accompanied by chronic low-grade inflammation,^[25,26]^ this pattern suggests that inflammatory status may be associated with sleep-disorder status.

In multivariable regression analysis, in contrast to existing studies,^[14,27,28]^ our study found no significant association between the SII and self-reported sleep disorders. Possible reasons include: we used self-reported sleep disorder as the outcome, a measure that conceptually encompasses multiple sleep disorder phenotypes and entails some nondifferential misclassification; combined with the single time-point assessment of SII and its short-term variability, this could attenuate the true association toward the null. Moreover, RCS analyses suggested a U-shaped relationship between SII and sleep, such that effects at the lower and higher ends offset each other under a linear specification. After comprehensive adjustment for metabolic comorbidities, the independent linear effect of SII on sleep disorder approached zero, yielding an overall nonsignificant association. Additionally, Walker et al, in a study including 392 nurses, found that inflammatory biomarkers were not associated with sleep disorders in fully adjusted models.^[29]^ Our findings are consistent with those of that study, likely because both analyses fully adjusted for covariates. After adjustment, the independent effect of SII on sleep disorders approached zero, yielding a nonsignificant association between SII and sleep disorders. Subsequent nonlinear analyses and subgroup analyses further revealed the complex relationship between SII and sleep disorders.

RCS analysis revealed a nonlinear association between SII and sleep disorders, with a U-shaped curve, indicating that both excessively high and low SII values were associated with a higher risk of sleep disorders. This is consistent with the findings of Yang et al both excessively long and excessively short durations of weekend catch-up sleep are associated with elevated SII,^[30]^ further supporting the overarching sleep–inflammation framework. Accordingly, the RCS curve shows a U-shaped pattern. On the one hand, this may be because when SII is elevated, neutrophils increase and become activated to release pro-inflammatory mediators; elevations in neutrophil and lymphocyte counts have also been linked to sleep disorders.^[31]^ In addition, higher platelet counts can promote leukocyte adhesion and recruitment which, together with inflammation-related endothelial dysfunction, facilitates sleep fragmentation.^[32]^ On the other hand, prior studies indicate that moderate levels of inflammation can promote non-rapid eye movement sleep, whereas excessive suppression of inflammatory activity diminishes the homeostatic drive to fall asleep and limits rebound sleep.^[18]^

In the subgroup analyses, interactions by race, education level, PIR, BMI, smoking status, and DM were not statistically significant, indicating that the findings are reasonably consistent and robust in the overall population. By contrast, the interactions for sex and HTN status were significant (*P* < .05), which may be explained as follows: this likely reflects the heavier burden and greater detectability of OSA among men.^[33]^ OSA is closely linked to neutrophil-predominant inflammation and platelet activation: both of which are captured by the SII. Prior studies show a higher prevalence of OSA in men; therefore, self-reported sleep disorders among men are more representative of OSA.^[34]^ In patients with OSA, the neutrophil-to-lymphocyte ratio and platelet activation are elevated, and men also exhibit sex-dimorphic immune responses that preferentially activate innate immune pathways.^[35]^ Taken together, these factors may strengthen the association between SII and sleep disorders in men. In hypertensive patients, these associations were attenuated. The widespread use of ACEIs/ARBs, statins, and antiplatelet agents in this group may lower systemic inflammatory mediators or platelet activity,^[36]^ thereby potentially reducing the variability of SII and weakening its subsequent association with sleep disorders.

To our knowledge, this is the first study to analyze the relationship between SII and sleep disorders using the latest NHANES database. This cross-sectional study suggests a co-occurrence between the SII and self-reported sleep disorders; however, after fully adjusting for age, sex, race, education, PIR, BMI, smoking, DM, CVD, and HTN, the linear association between SII and sleep disorders was no longer significant, and the overall relationship may be nonlinear. Accordingly, SII is better suited in clinical practice as an auxiliary cue within a comprehensive assessment rather than a standalone basis for determination: when encountering clues such as snoring, daytime sleepiness, difficulty initiating or maintaining sleep, and morning headache, priority should be given to combining standardized scales and, when necessary, considering objective monitoring or referral, while focusing management on co-occurring, modifiable factors such as weight management, blood pressure and glucose control, smoking cessation, psychological and stress management, and sleep-hygiene education. Stratified results indicate that this co-occurrence is more evident in men and in individuals without HTN, in whom screening sensitivity may be modestly increased. It is important to note that although the effect size of SII is small and its incremental value for individual decision-making is limited, it remains practically useful for “sentinel surveillance (early warning) resource allocation” in large-scale population monitoring and primary public-health settings, where it can be combined with age, sex, BMI, and smoking history to form simple alert rules and be used alongside existing scales to optimize education and screening coverage and to track population-level trends. At the same time, attention should be paid to the boundaries of the evidence: outcomes are self-reported, SII was measured once, nonlinear thresholds are undetermined, and the cross-sectional design limits temporal inference; clinical application should adhere to the principle of “comprehensive assessment (prudent decision-making) evidence-based iteration,” avoiding over-interpretation of a single inflammatory marker.

## 5. Study strengths and limitations

The main strength of this study lies in the use of the nationally representative NHANES database, with a large sample size and high population heterogeneity, which enhances the generalizability of the results. Various confounding factors, including demographics, socioeconomic status, and metabolic diseases, were adjusted using multivariable models. Additionally, the RCS model was employed to reveal the nonlinear dose–response relationship between SII and sleep disorders, with the RCS curve showing a U-shape, suggesting the possibility of a threshold effect. Subgroup analysis found that the effect of SII on sleep disorders was more significant in males, which may be related to genetic background or differences in lifestyle. This phenomenon warrants further exploration in future studies.

However, this study has several limitations. First, the diagnosis of sleep disorders was based on self-reported data from patients rather than sleep monitoring or clinical assessment, which may introduce misclassification bias. Second, although various confounding factors were adjusted for, residual confounding may still affect the results. Third, the cross-sectional design cannot establish causality, and prospective cohort studies or randomized controlled trials are needed for validation. Additionally, the NHANES data only represent the U.S. population, so extrapolating the conclusions to other ethnic groups or regions should be done with caution. Future research should incorporate polysomnography and imaging biomarkers to further clarify the temporal role of SII in the pathological progression of sleep disorders.

## 6. Conclusion

This cross-sectional analysis of NHANES 2005 to 2020 found a positive association between SII and sleep disorders in unadjusted models. However, after adjusting for potential confounders, the association was no longer statistically significant, suggesting that the observed relationship may largely reflect shared correlates rather than an independent link with SII. Accordingly, in populations with higher SII, it may be reasonable to prioritize screening for metabolic abnormalities and addressing modifiable coexisting factors within a comprehensive assessment, rather than relying on inflammatory markers alone to manage sleep complaints. Future high-quality longitudinal studies are needed to clarify temporal patterns and evaluate the incremental clinical utility of SII in sleep health.

## Author contributions

**Conceptualization:** Yang Yang, Defeng Chen, Yueyu Liang.

**Data curation:** Yang Yang, Defeng Chen, Bei Li.

**Formal analysis:** Yang Yang, Defeng Chen.

**Funding acquisition:** Defeng Chen.

**Investigation:** Yang Yang.

**Methodology:** Yang Yang.

**Project administration:** Yang Yang.

**Resources:** Yang Yang.

**Software:** Yang Yang, Jiabao Li.

**Supervision:** Yang Yang, Xuhui Dong.

**Validation:** Yang Yang.

**Visualization:** Yang Yang.

**Writing – original draft:** Yang Yang, Defeng Chen.

**Writing – review & editing:** Yang Yang, Defeng Chen, Xuhui Dong, Mengxin He, Yaokai Xu.
